# Draft Genome Sequences of Three Filamentous Cyanobacterial Strains, Dolichospermum planctonicum NIES-80, Planktothrix agardhii NIES-905, and Sphaerospermopsis reniformis NIES-1949

**DOI:** 10.1128/MRA.00605-19

**Published:** 2019-10-24

**Authors:** Shigekatsu Suzuki, Haruyo Yamaguchi, Masanobu Kawachi

**Affiliations:** a Center for Environmental Biology and Ecosystem Studies, National Institute for Environmental Studies, Tsukuba, Japan; University of Delaware

## Abstract

Three freshwater planktonic filamentous cyanobacterial strains, Dolichospermum planctonicum NIES-80, Planktothrix agardhii NIES-905, and Sphaerospermopsis reniformis NIES-1949, were sequenced. The genome sizes of NIES-80, NIES-905, and NIES-1949 were 4,571,002 bp, 5,512,454 bp, and 6,025,023 bp, and the number of protein-coding genes in each genome was 4,009, 4,925, and 5,408, respectively.

## ANNOUNCEMENT

Water blooms, also called “cyanobacterial blooms” (1), are mainly formed by some planktonic cyanobacterial species in mesotrophic-eutrophic freshwater (2). Among these species, filamentous cyanobacteria have ecological importance. They are the main primary producers and secondary metabolite producers (e.g., cyanotoxins) and are also involved in nitrogen fixation (3). We sequenced the genomes of three bloom-forming filamentous cyanobacterial strains, Dolichospermum planctonicum NIES-80, Planktothrix agardhii NIES-905, and Sphaerospermopsis reniformis NIES-1949.

All three axenic strains were maintained at the National Institute for Environmental Studies (NIES). NIES-80 and NIES-1949 were cultured in 10 ml of CB medium at 22°C and 20°C, respectively. NIES-905 was cultured in 10 ml of CT medium (4) at 20°C. Cyanobacteria were cultured for 3 weeks and harvested using gentle centrifugation. DNA was extracted from NIES-80 and NIES-905 using a DNeasy plant minikit (Qiagen, Düsseldorf, Germany), while NIES-1949 DNA extraction was performed using an Agencourt Chloropure kit (Beckman Coulter, Brea, CA) per the manufacturer’s instructions. DNA was sheared to a fragment size of approximately 550 bp using an M220 focused ultrasonicator (Covaris, Woburn, MA). The DNA libraries were constructed using a NEBNext Ultra II DNA library prep kit for Illumina (New England Biolabs, Ipswich, MA). The libraries were sequenced on the MiSeq platform (Illumina, San Diego, CA) using a MiSeq reagent kit v3. We sequenced 587,952,677 bp (NIES-80), 750,851,958 bp (NIES-905), and 125,087,525 bp (NIES-1949) paired-end reads. The raw reads were trimmed using Trimmomatic v0.38 (5) and assembled using SPAdes v3.11.1 (6) and Shovill v1.0.4 (https://github.com/tseemann/shovill). The assemblies were polished using Pilon v1.22 (7). After removal of the short reads (<200 bp), gene model construction and functional annotation were performed using the DFAST legacy server (8) and CyanoBase (9).

The draft genome sizes of NIES-80, NIES-905, and NIES-1949 were 4,571,002 bp (GC content, 37.7%), 5,512,454 bp (GC content, 39.5%), and 6,025,023 bp (GC content, 37.5%), respectively (Table 1). The genome completeness rates were 99.22% (NIES-80), 100.00% (NIES-905), and 98.89% (NIES-1949), respectively, as evaluated by CheckM analysis (10). The number of protein-coding genes in the genomes of NIES-80, NIES-905, and NIES-1949 was 4,009, 4,925, and 5,408, respectively. The genomes of NIES-80, NIES-905, and NIES-1949 were similar to those of Dolichospermum compactum NIES-806, *P. agardhii* NIES-204, and Sphaerospermopsis kisseleviana NIES-73, respectively, in terms of size and the number of genes. Some strains of Planktothrix agardhii produce a cyanotoxin, microcystin, that is synthesized by the *mcy* gene cluster (11). The NIES-905 sequence included a conserved *mcy* gene cluster (*mcyJ*, *mcyC*, *mcyB*, *mcyA*, *mcyH*, *mcyG*, *mcyE*, and *mcyD*), suggesting that this strain can probably synthesize microcystin. The three species used in this study are widely present in freshwater. Thus, the genomes can be used for monitoring using quantitative PCR or droplet digital PCR (12) and for metagenomic analyses of cyanobacterial blooms.

**TABLE 1:** Genome features of NIES-80, NIES-905, and NIES-1949

Species	Strain	Assembly size (bp)	No. of contigs	*N*_50_ (kbp)	Genome completeness (%)	GC content (%)	No. of protein-coding genes	No. of tRNAs	No. of rRNAs
*Dolichospermum planctonicum*	NIES-80	4,571,002	201	75.2	99.22	37.70	4,009	44	7
Planktothrix agardhii	NIES-905	5,512,454	193	93.2	100.00	39.50	4,925	40	5
*Sphaerospermopsis reniformis*	NIES-1949	6,025,023	680	20.0	98.89	37.50	5,408	43	2

### Data availability.

The draft genome sequences of *Dolichospermum planctonicum* NIES-80, Planktothrix agardhii NIES-905 (= CCAP 1459/11A), and *Sphaerospermopsis reniformis* NIES-1949 have been deposited in DDBJ/EMBL/GenBank under the accession numbers BJCF01000001 to BJCF01000201, BJCD01000001 to BJCD01000193, and BJCE01000001 to BJCE01000680, respectively. The genomic raw reads are also available in DDBJ/EMBL/GenBank under the accession numbers DRR172254, DRR172255, and DRR172253, respectively.
